# HIV-1 replicates and persists in vaginal epithelial dendritic cells

**DOI:** 10.1172/JCI98943

**Published:** 2018-07-09

**Authors:** Victor Pena-Cruz, Luis M. Agosto, Hisashi Akiyama, Alex Olson, Yvetane Moreau, Jean-Robert Larrieux, Andrew Henderson, Suryaram Gummuluru, Manish Sagar

**Affiliations:** 1 Department of Medicine and; 2 Department of Microbiology, Boston University School of Medicine, Boston, Massachusetts, USA.; 3 Department of Obstetrics and Gynecology, Boston University, Boston, Massachusetts, USA.

**Keywords:** AIDS/HIV, Infectious disease, Dendritic cells

## Abstract

HIV-1 acquisition occurs most commonly after sexual contact. To establish infection, HIV-1 must infect cells that support high-level replication, namely CD4^+^ T cells, which are absent from the outermost genital epithelium. Dendritic cells (DCs), present in mucosal epithelia, potentially facilitate HIV-1 acquisition. We show that vaginal epithelial DCs, termed CD1a^+^ VEDCs, are unlike other blood- and tissue-derived DCs because they express langerin but not DC-SIGN, and unlike skin-based langerin^+^ DC subset Langerhans cells (LCs), they do not harbor Birbeck granules. Individuals primarily acquire HIV-1 that utilizes the CCR5 receptor (termed either R5 or R5X4) during heterosexual transmission, and the mechanism for the block against variants that only use the CXCR4 receptor (classified as X4) remains unclear. We show that X4 as compared with R5 HIV-1 shows limited to no replication in CD1a^+^ VEDCs. This differential replication occurs after fusion, suggesting that receptor usage influences postentry steps in the virus life cycle. Furthermore, CD1a^+^ VEDCs isolated from HIV-1–infected virologically suppressed women harbor HIV-1 DNA. Thus, CD1a^+^ VEDCs are potentially infected early during heterosexual transmission and also retain virus during treatment. Understanding the interplay between HIV-1 and CD1a^+^ VEDCs is important for future prevention and cure strategies.

## Graphical abstract

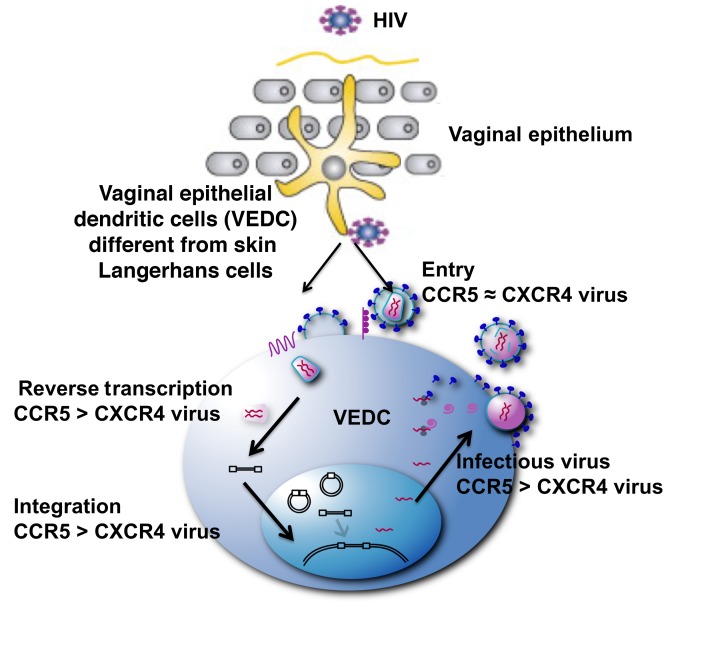

## Introduction

The majority of new HIV-1 infections occur in women after heterosexual contact. To establish a systemic infection in a naive woman, HIV-1 must cross the genital epithelium and infect cells that support high-level replication, namely CD4^+^ T cells. A dendritic cell (DC) subset previously classified as Langerhans cells (LCs) is generally present in the outermost genital epithelial layers, but CD4^+^ T lymphocytes are not (1). This anatomical distribution and previous characterization suggests that access to the deeper-lying CD4^+^ T cells potentially occurs as a result of cell-to-cell transfer from these epithelial-based DCs (2). Skin-based LCs are often deemed representative of the DCs present in genital epithelia. Skin-based LCs express the CD4 receptor and a coreceptor, either CCR5 or CXCR4, required for HIV-1 entry (3). Skin-based LCs, however, have never been shown to harbor HIV-1 DNA in vivo, possibly because they express langerin and harbor Birbeck granules (BGs), which protect against HIV-1 infection (4, 5). This block against HIV-1 occurs against both strains that utilize the CCR5 receptor, termed either R5 or R5X4, and the variants that exclusively use the CXCR4 receptor, classified as X4. Vaginal epithelial DCs (VEDCs), however, must have unique characteristics as compared with skin-based LCs because HIV-1 is commonly acquired across mucosal surfaces but not from exposed skin. We show that VEDCs as compared with skin LCs lack BGs, possibly explaining their susceptibility to HIV-1 infection. Furthermore, R5 as compared with X4 viruses preferentially replicate in VEDCs, and factors present after host cell entry influence this differential replication. This suggests that VEDCs are important for the selection that occurs during HIV-1 acquisition because the majority of new infections occur with viruses that use the CCR5 receptor as opposed to X4 strains (6). We also demonstrate that VEDCs from infected virologically suppressed women have HIV-1 DNA, suggesting that these cells are infected in vivo. Thus, the unique VEDCs possibly represent a previously unrecognized viral reservoir.

## Results and Discussion

**Figure 1:**
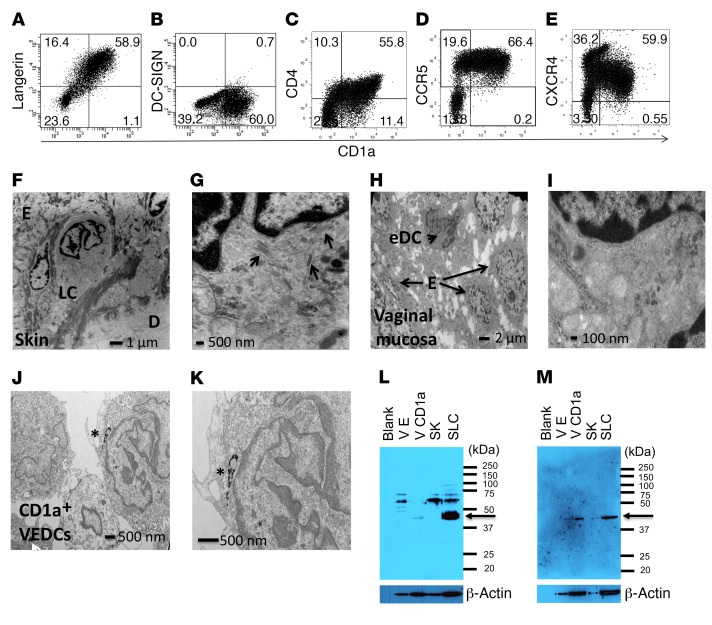
Vaginal CD1a^+^ cells are a unique DC subset. (**A**–**E**) Representative dot plots from a minimum of 3 independent donors show staining for CD1a along with (**A**) Langerin (CD207) , (**B**) DC-SIGN (CD209), (**C**) CD4, (**D**) CCR5, and (**E**) CXCR4. Numbers in the quadrants show the percentage of positive cells. Due to limited cell quantities, the CD1a^+^ VEDCs in these plots are not all from the same tissue. (**F** and **G**) Electron micrograph (EM) of the skin with markers denoting epithelium (E), Langerhans cell (LC), and dermis (D). The arrows point at morphological structures consistent with Birbeck granules (BG). (**H** and **I**) EM of vaginal tissue demonstrating epithelium (E) and a nucleated cell consistent with an epithelial-based dendritic cell (eDC). (**J** and **K**) EM of CD1a^+^ VEDC pellets with asterisks showing the CD1a beads. (**L** and **M**) Two independent Western blots of cell pellets from different vaginal tissue and skin donors. The vaginal epithelial (VE), vaginal CD1a^+^ cells (V CD1a), skin epithelial (SK), and skin Langerhans cells (SLC) were probed with Lag antibody (Takara), which is deemed specific for BGs. Expected band for BG binding is at 43 kDa, shown by arrow. Bottom blot shows probing for beta-actin.

To understand how HIV-1 acquisition could occur when CD4^+^ T cells are absent from the outermost nonulcerated genital layers, we cleanly separated the epithelium from vaginal lamina propria (Supplemental Figure 1; supplemental material available online with this article; https://doi.org/10.1172/JCI98943DS1). Thus, the subsequent single-cell isolations from the epithelia were not contaminated by contents from the lamina propria. We used previously described discontinuous density gradients (7) and magnetic bead–conjugated antibodies specific for a DC-specific marker (CD1a) to isolate epithelial-based DCs. A significantly lower number of CD1a^+^ VEDCs as compared with skin LCs was isolated from vaginal tissue as compared with skin (Supplemental Figure 2). Classically, the skin LCs express the C-type lectin receptor langerin, and not the classic DC cell surface marker DC-SIGN (Supplemental Figure 3). A majority of CD1a^+^ VEDCs also expressed langerin (Figure 1A) and lacked DC-SIGN (Figure 1B), suggesting that these epithelial-based cells are distinct from the subepithelial-based DC-SIGN^+^ vaginal myeloid DCs (1, 8). A majority of the CD1a^+^ VEDCs also expressed CD4, CCR5, and CXCR4 (Figure 1, C–E and Supplemental Figure 4). The presence and absence of other markers suggested that the CD1a^+^ epithelial cell isolations were devoid of tissue macrophages (9) (Supplemental Figure 4) and lymphocytes (Supplemental Figure 5), and the cells were mostly in an inactive state (Supplemental Figure 6).

Electron microscopy (EM) of skin cells in situ clearly demonstrated cytoplasmic BGs, a hallmark of all LCs (Figure 1, F and G) (10). In contrast, a minimum of 10 separate fields each in vaginal tissue from 5 different donors revealed no morphological structure resembling BGs (Figure 1, H and I). EM examination of purified CD1a^+^ cell pellets showed lobulated nucleus and projecting dendrites, but BGs were not evident (Figure 1, J and K). Western blots demonstrated that vaginal epithelial CD1a^+^ cells compared with skin LCs had minimal amount of a protein that bound an antibody (Lag) deemed specific for BGs (Figure 1, L and M) (11, 12). In contrast to in vitro studies (11, 13, 14), our observations suggest that langerin expression does not lead to the presence of classic BGs in the CD1a^+^ VEDCs. Similarly, classic BGs have also not been observed in murine vaginal epithelial presumed LCs (15). Thus, CD1a^+^ VEDCs are a unique, previously undefined human DC subset because unlike monocyte-derived DCs (MDDCs) or vaginal subepithelial DCs, they express langerin and not DC-SIGN and unlike skin-derived LCs they lack BGs.

**Figure 2:**
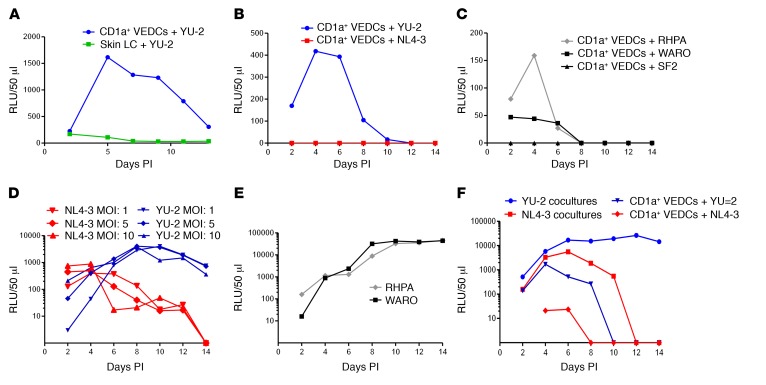
R5 and X4 HIV-1 have differential replication in CD1a^+^ VEDCs. Each graph shows relative light units (RLU) (*y* axis) generated from TZM-bl cells 48 hours after being exposed to 50 μl of culture supernatant, which was collected days after infection (PI) (*x* axis). Days PI was defined as either days after virus-exposed cells were washed to remove unbound virus or the start of coculture. Replication of YU-2 (R5; MOI: 15), NL4-3 (X4; MOI: 15), transmitted/founder (RHPA; MOI: 10), chronic infection strain (WARO; MOI: 10), and SF2 (X4; MOI: 8) in (**A**) CD1a^+^ VEDCs and skin-derived LCs, (**B**, **C**, **F**) CD1a^+^ VEDCs, (**D** and **E**) vaginal tissue resident lymphocytes, and (**F**) CD1a^+^ VEDCs cocultured with autologous vaginal tissue resident lymphocytes. For each graph, the CD1a^+^ VEDCs were obtained from a different individual’s tissue. Each plotted RLU is the amount above background, and any RLU value below background was assigned a value of 0. RLUs observed at day 2 PI do not reflect residual virus from inocula (see Supplemental Figure 11).

Previous investigations have suggested that skin LCs internalize HIV-1 using langerin and degrade internalized virus in BGs, although virus challenges initiated at high multiplicity of infection (MOI) can overcome this block (4, 5). Similar to our previous report with other primary strains (16), HIV-1 isolate YU-2, which requires the CCR5 coreceptor for cell entry, did not replicate in skin-derived LCs even when exposed to high MOIs (Figure 2A). In contrast, YU-2 established a low-level spreading infection in CD1a^+^ VEDCs from different donors (Figure 2, A, B, and F, and Supplemental Figure 7). No infectious virus, however, was observed in the CD1a^+^ VEDCs exposed to similarly high MOIs of exclusive CXCR4-using viruses NL4-3 and SF2 (Figure 2, B and C). CD1a^+^ VEDCs also supported replication of a CCR5-dependent infectious molecular clone (IMC) (RHPA) isolated from an individual during the acute phase of infection, termed a transmitted/founder (T/F) variant (Figure 2C) (17). The RHPA–CD1a^+^ VEDC cultures yielded nearly 3-fold more infectious viruses at day 4 after infection compared with another primary CCR5-using IMC isolated from a heterosexually infected woman during chronic infection (WARO) (Figure 2C) (17). Thus, R5 variants (including a T/F strain) but not X4 viruses replicated in CD1a^+^ VEDCs and not in skin-derived LCs.

As opposed to the differential growth observed in the CD1a^+^ VEDCs, YU-2, NL4-3, RHPA, and WARO replicated in activated cells from the lamina propria (Figure 2, D and E), which are primarily tissue-resident lymphocytes (TRLs) (Supplemental Figure 8) (18). Furthermore, both NL4-3 and YU-2 replicated in virus-exposed and subsequently washed CD1a^+^ VEDCs cocultured with autologous activated TRLs (Figure 2F). In aggregate, R5 as compared with X4 variants had differential replication in CD1a^+^ VEDCs alone but not in activated vaginal TRLs cocultured with or without CD1a^+^ VEDCs.

**Figure 3:**
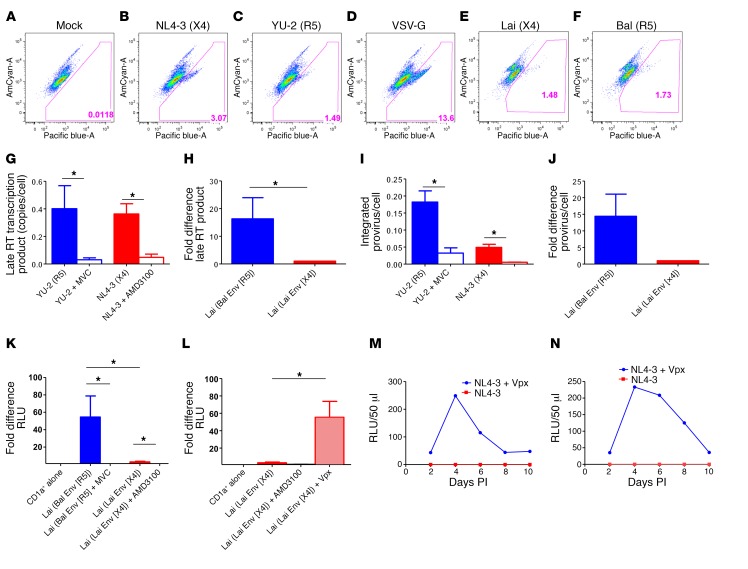
Limitation in X4 variant replication occurs after entry. (**A**–**F**) Fusion observed in CD1a^+^ VEDCs that were (**A**) mock infected or exposed to pseudovirions with (**B**) NL4-3 (X4), (**C**) YU-2 (R5), (**D**) VSV-G (positive control), (**E**) Lai, and (**F**) Bal envelope. Numbers at the bottom show the percentage of fusion. (**G**–**J**) Late reverse transcription products (**G** and **H**) and integrated provirus (**I** and **J**) in CD1a^+^ VEDCs among R5 (blue) and X4 (red) envelope viruses in the absence and presence of CCR5 blocker Maraviroc (MVC) (blue outline) and CXCR inhibitor AMD3100 (red outline). Experiments were done with replication-competent infectious molecular clones YU-2 (R5) and NL4-3 (X4) (*n* = 3 tissues; comparisons used a 2-sided *t* test) (**G** and **I**) or a single-cycle reporter virus pseudotyped with either a CCR5-using (Bal) or CXCR4-using (Lai) envelope (*n* = 7 tissues; comparisons used a 2-sided Wilcoxon signed rank test with Lai set as the reference) (**H** and **J**). (**K**) Fold difference in luciferase expression in CD1a^+^ VEDCs (*n* = 7 tissues) 3 days after exposure to either media alone (set as reference), Lai/Bal (R5), or Lai/Lai (X4) reporter pseudotypes in the presence and absence of entry inhibitors (comparisons used a 2-sided Wilcoxon signed rank test). (**L**) Fold difference in luciferase expression in CD1a^+^ VEDCs (*n* = 4 tissues) 3 days after exposure to either media alone (set as reference) or Lai/Lai (X4) in the presence or absence of entry inhibitor and SIV Vpx (light red shading)(comparison with and without Vpx done with a 2-sided Mann-Whitney *U* test). (**M** and **N**) RLUs generated from TZM-bl cells 48 hours after being exposed to virus supernatants from CD1a^+^ VEDCs exposed to NL4-3 or NL4-3 in the presence of SIV Vpx. *P < 0.05.

In contrast to skin LCs, the X4 variants’ poor replication in CD1a^+^ VEDCs is not due to the absence of the CXCR4 receptor (Figure 1E and Supplemental Figure 4) (3). Indeed, X4 variants fuse with CD1a^+^ VEDCs at a level similar to that of R5 variants (Figure 3, A–F and Supplemental Figure 9). This phenotype is dramatically different from MDDCs, to which R5 virus fuses at a significantly higher level compared with an X4 variant (Supplemental Figure 10). Both X4 and R5 envelope strains complete reverse transcription and integration in the CD1a^+^ VEDCs (Figure 3, G–J). In CD1a^+^ VEDCs an R5 as compared with an X4 envelope virus within an isogenic backbone, however, demonstrated higher reverse transcription (mean fold difference 8.2, range 1.1–22.8, *n* = 7, *P* = 0.02) and integration (mean fold difference 10.1, range 0.7–26.7, *n* = 7, *P* = 0.30) (Figure 3, H and J). Viral gene transcription was significantly higher in the absence than in the presence of coreceptor blockers in CD1a^+^ VEDCs for both R5 and X4 pseudoviruses (Figure 3K). Thus, after integration, transcription occurs with both types of viruses. Importantly, luciferase expression (mean fold difference 23.9, range 2.2–104.2, *n* = 7, *P* = 0.02) was higher among CD1a^+^ VEDCs exposed to the R5 as compared with the X4 envelope virus within an isogenic backbone (Figure 3K). Thus, viral envelope host receptor interactions influence the virus postentry life cycle in CD1a^+^ VEDCs.

Host restriction factor SAMHD1 inhibits HIV-1 reverse transcription and subsequent integration in myeloid cells (19, 20). However, the SIV and HIV-2 accessory protein Vpx can alleviate this block by promoting SAMHD1 degradation (Supplemental Figure 11) (19, 20). CD1a^+^ VEDCs expressed similar levels of total SAMHD1 and the inactive phosphorylated form of SAMHD1 after exposure to media alone or virus (Supplemental Figure 11). Luciferase expression was higher (mean fold difference 23.2, range 8.3–59.7, *n* = 4, *P* = 0.03) in CD1a^+^ VEDCs in the presence than in the absence of SIV Vpx for an X4 virus (Figure 3L). HIV-1 X4 virus replication was also observed in the presence but not the absence of SIV Vpx in CD1a^+^ VEDC cultures (Figure 3, M and N). Presence of SIV Vpx did not impact replication in cells from the lamina propria or in CD1a^+^ VEDCs exposed to YU-2 (Supplemental Figure 11). In aggregate, this demonstrates that SAMHD1 also restricts HIV-1 replication in CD1a^+^ VEDCs.

**Figure 4:**
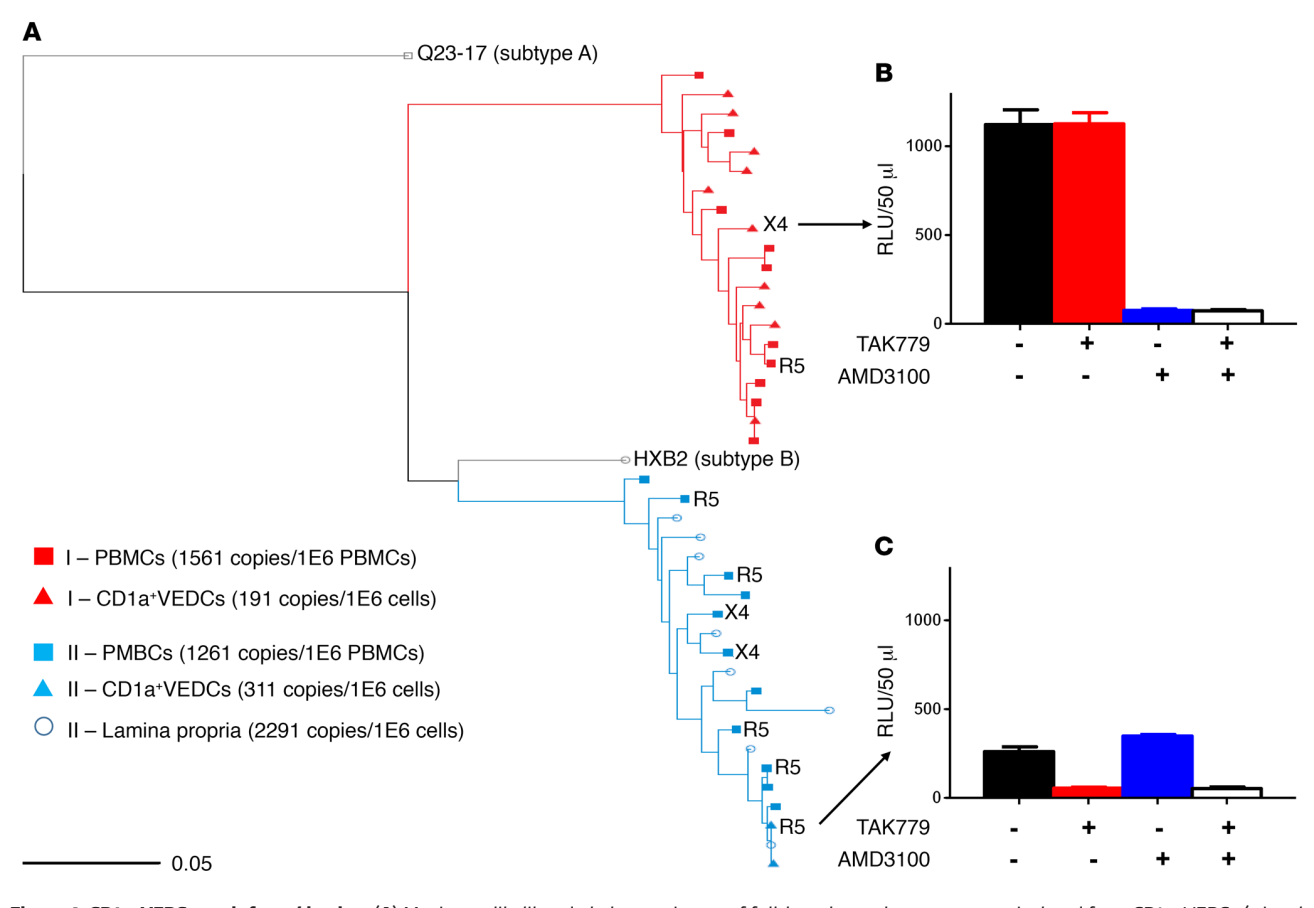
CD1a^+^ VEDCs are infected in vivo. (**A**) Maximum likelihood phylogenetic tree of full-length envelope sequences isolated from CD1a^+^ VEDCs (triangles), peripheral blood mononuclear cells (squares), and the lamina propria (open circles). Sequences from each subject are denoted by different colors. Number of HIV-1 copies estimated per million cells is indicated in the key. The phenotypically determined receptor usage of some of the virus stocks incorporating the isolated envelopes with a HIV-1 NL4-3 backbone is denoted next to a node as either X4 or R5. The Q23-17 (subtype A) outgroup and the NL4-3 (subtype B) nodes are also identified. (**B** and **C**) RLUs after 48 hours in TZM-bl cells exposed to virus stocks incorporating the CD1a^+^ VEDC–isolated envelopes in the presence of no inhibitor (black), TAK779 (red), AMD3100 (blue), and both TAK779 and AMD3100 (white). Bars show mean with SEM of infections done in triplicate.

Contemporaneous vaginal tissue and blood samples were obtained from 2 HIV-1–infected virologically suppressed women to provide evidence that CD1a^+^ VEDCs are infected in vivo. Averages of 5.0 and 3.7 HIV-1 DNA copies were detected in means of 16,136 (311 copies/10^6^) and 19,523 (191 copies/10^6^) CD1a^+^ VEDCs from woman I and woman II, respectively (Supplemental Table 1). In comparison, provirus copy numbers were around 4- to 8-fold higher in peripheral blood mononuclear cells (PBMCs) (1,261 and 1,561 copies/10^6^ in woman I and woman II, respectively) and in lamina propria cells (2,291 copies/10^6^ in woman II and data not available from woman I). HIV-1 DNA was below 1 copy per 10,000 cells from the CD1a^–^ vaginal epithelial fraction in both individuals. Single genome amplification revealed that full-length envelope sequences from the CD1a^+^ VEDCs, PBMCs, and cells in the lamina propria were intermingled, suggesting these cells harbored viruses from a similar ancestral stage of infection (Figure 4A). Incorporation of the isolated CD1a^+^ VEDC and PBMC envelopes into an envelope-deficient NL4-3 backbone yielded both replication-competent R5 and X4 virus stocks (Figure 4, B and C). Thus, CD1a^+^ VEDCs harbor HIV-1 DNA with functional X4 and R5 envelopes, suggesting they are infected with viruses that use either receptor in vivo.

In this study, we isolated vaginal epithelial-based cells that are most likely to encounter virus in the female genital tract. We have shown that the CD1a^+^ VEDCs are not analogous to classically defined skin LCs as previously presumed (1, 2, 21, 22), and that they are different from other subepithelial and blood-derived DCs. In some respects, our findings agree with mouse models showing that vaginal epithelial-based DCs are phenotypically different from skin-derived LCs (15, 23). In contrast to previous studies, we showed that CD1a^+^ VEDCs either do not contain or have low levels of BGs, and thus they cannot be characterized as LCs but are rather a unique previously undefined human DC subset. Lack of BGs potentially explains the difference in susceptibility to infection among CD1a^+^ VEDCs as compared with skin LCs (4, 5).

We have also demonstrated that CD1a^+^ VEDCs support higher replication of R5 compared with X4 HIV-1. This potentially explains the epidemiological observation that the majority of mucosally acquired infecting strains utilize the CCR5 receptor (6). In contrast to other studies (21, 22), our work suggests that the limited replication of X4 viruses occurs from differential replication in the CD1a^+^ VEDCs and is not due to attenuated replication in or cell-to-cell transfer to activated TRLs. Similar to a previous study, de novo virus production after fusion occurs intermittently, which suggests that after entry there are both receptor-independent blocks, such as SAMHD1, and other potentially novel receptor-dependent barriers (24). Although presence of HIV-1 DNA in CD1a^+^ VEDCs from infected women confirms in vivo infection, future studies will need to show that the CD1a^+^ VEDCs that harbor HIV-1 DNA can yield replication-competent virus and that the DNA does not merely represent engulfed infected CD4^+^ T cells (25). In aggregate, CD1a^+^ VEDCs are most likely the initial “gatekeeper” that selects viruses that will successfully establish an infection in a naive woman. Furthermore, virus persists in these cells during effective antiretroviral treatment, and thus, CD1a^+^ VEDCs may be a previously unrecognized latent reservoir.

## Methods

Please see the Supplemental Methods for a detailed explanation of all experimental procedures.

### Study approval.

Genital and breast tissue and blood sample acquisition was approved by the IRB at Boston University and Brigham and Women’s Hospital. Women who provided contemporaneous blood and genital samples gave written informed consent, and the remaining tissues were obtained as discarded surgical samples.

### Statistics.

All comparisons were done using 2-tailed Student’s *t* test, Wilcoxon signed rank test, or Mann-Whitney *U* test in GraphPad Prism 5. *P* < 0.05 was considered significant.

## Author contributions

VPC and MS designed the research studies and analyzed the data. VPC isolated CD1a^+^ VEDCs. VPC, LMA, HA, AO, and YM performed experiments. JRL provided clinical samples. AH and SG provided input regarding data interpretation. MS wrote the manuscript with input from the other authors.

## Supplementary Material

Supplemental data

## Version 1 05/03/2018

In-Press Preview

## Version 2 07/09/2018

Electronic publication

## Version 3 08/01/2018

Print issue publication
