# Supplementary material for: HIV-1 replicates and persists in vaginal epithelial dendritic cells
Source: J Clin Invest. 2018 Jul 9;128(8):3439–44. doi: 10.1172/JCI98943 (PMC6063466; doi:10.1172/JCI98943)
Supplement: Supplemental data [file jci-128-98943-s168.pdf]

Methods

Cell Isolation. Fresh genital and mammoplasty tissue was acquired as discarded surgical material after approval by the Institutional Review Boards (IRB) at Boston University and Brigham and Women's Hospital. CD1a+ cells were isolated using previously described methods with modifications (1). Briefly, adipose and connective tissue was removed from the epidermis, and the epithelium was mechanically separated from the sub-epithelium after overnight incubation in dispase II. The epithelium was further digested in trypsin and individual cells were obtained after filtration. Epithelial cells were passaged through a discontinuous OptiPrep density gradient, and magnetic CD1a microbeads were used to isolate CD1a+ cells (Miltenyi Biotech). VEDCs were maintained in RPMI complete containing 10% fetal bovine serum, 2mM L-glutamine, 100U of penicillin per ml, and 100 μg of streptomycin per ml.

Activated and non-activated lamina propria cells were obtained by collecting migratory cells from the sub-epithelial tissue that was incubated in RPMI complete either supplemented with or lacking 2% phytohaemagglutinin (PHA; Invitrogen) respectively for 3 days. In both cases, cells were filtered, and lymphocytes were collected using a discontinuous OptiPrep density gradient. Activated and non-activated cells were cultured in RPMI complete with 20U/ml recombinant human IL-2 (r-IL2) respectively.

Flow Cytometry. Flow cytometry was performed using a BD FACSCanto II and analyzed on BD FACSDiva software (Becton Dickinson); and on a Cytomics FC500 (Beckman Coulter) and analyzed with FlowJo software (Tree Star) or Flowing Software (version 2.5.1).

Antibodies. The following antibodies were used for flow cytometric analysis: CD1a FITC Clone HI149 (Thermo Scientific), CD1a Fluor 450 Clone HI149, CD8 PerCPcy 5.5 Clone RPA-T8, CD45-FITC Clone 2D1, and CD4-APC Clone RPA T-4 (eBioscience), CD207 PE Clone DCGM4 (Immunotech), CD11c PE Clone B-ly6 (RUO) (BD Biosciences), DC-SIGN FITC Clone DCN46, CD69-PE Clone FN50, CD4-PE Clone OKT-4, CD80-FITC Clone HB15a, CD86-APC Clone 2331, CD86 APC Clone 2331, CD14-FITC Clone M5E2, and CD19-PE Clone HIB19 (BD

Pharmigen), CD4-PerCp Cy5.5 Clone RPA T-4, CCR5-PE Clone J418F1, CD3-FITC Clone VCHT1, and CXCR-PE Clone 12G5 (Biolegend).

Western blot. Whole cell extracts were prepared by suspending cells in lysis buffer (10 mMTris-Cl (pH 7.4), 150 mM NaCl, 1.0 mM EDTA (pH 8.0), 2.0 mM sodium vanadate, 10 mM sodium fluoride, 10 mM sodium pyrophosphate, 1% Triton X 100, 1.0 mM phenylmethylsulfonyl fluoride, and protease inhibitor mixture III (Calbiochem)). The cell lysate were suspended in SDS-PAGE loading buffer, and heated for 5 min at 100 °C before resolving on 12% SDS-PAGE along with relevant controls and pre-stained protein markers. Separated proteins were transferred (at 100V for 1.5 hour at 4°C) onto a nitrocellulose membrane using the Mini Trans-blot apparatus (Bio-Rad Laboratories). Blots were incubated in blocking buffer in 5% non-fat milk (NFM) for 2 hours, and blotted overnight with anti-Birbeck granule monoclonal antibody, Lag clone 4D12, (IBL Co.) at concentration (1:5000) in 1× PBS, 0.05% Tween 20 (1× PBS-T) (3). The blots were washed again with 1× PBS-T (three washes, 10 minutes/wash), and incubated with rabbit generated polyclonal anti-mouse IgG-HRP secondary antibody (Sigma-Aldrich) at concentration (1:10000) for 45 minutes at room temperature. The blots were washed again as above and were treated with Super Signal West Pico Chemiluminescent HRP substrate (ECL) HRP reagent (Thermo Scientific) for 5 min.

To assess expression of SAMHD1, whole cell extracts were separated by SDS-PAGE, transferred to nitrocellulose membranes and the membranes were probed with either a mouse polyclonal anti-SAMHD1 antibody (Abcam), rabbit anti SAMHD1 (Cell Signaling #12361), or rabbit anti-phosphorylated-SAMHD1 (Cell Signaling Technology) followed by a goat anti-mouse-IgG-IRDye 680 (Pierce) or a goat anti-rabbit-IgG-IRDye 800CW (Pierce). As loading controls, actin was probed either using rabbit anti-actin (SIGMA) or mouse anti actin (ThermoFisher #AM4302). The membranes were scanned with an Odyssey scanner (Li-Cor).

Electron microscopy (EM). Tissues and cells were examined as described previously (1). Briefly, formaldehyde fixed tissue was treated with osmium in 0.085 M cacodylate buffer,

dehydrated using increasing hexamethyl disilazane (HMDS) (Electron Microscope Sciences) concentrations in ethanol. Vacuum dried tissue was sputter coated with metal (Au/Pd in a Hummer V, Anatech) and observed under the scanning electron microscope (Amray). The mucosa and cell pellets were fixed at room temperature in 0.1M cacodylate buffer (pH 7.4) containing formaldehyde 2.5%, glutaraldehyde 5% and picric acid 0.06% (Aldrich). The sample was embedded in epon araldite, 2.4 volumes of dodecinyl succinic anhydride and propylene oxide and hardened to 60ºC. Sections were examined with a JEM-1200 EX electron microscope (JEOL).

Viruses. Plasmids containing infectious molecular clones (IMC) (YU-2, NL4-3, RHPA, and WARO), expression plasmid (VSV-G, CCR5-tropic HIV gp160 (Bal Envelope), and the CXCR4-tropic HIV gp160 (Lai Envelope)), pMM310, containing the BlaM-Vpr construct, HIV-1 Lai∆env-luc (Envelope deficient HIV-1 Lai containing a luciferase reporter gene in place of the nef), and pSIV3^{+} (SIV$_{mac239}$ Vpx encoding SIV packaging vector) were obtained from the NIH AIDS Reference Reagent Program or described previously (4-6). SF2 virus stocks were obtained from NIH AIDS Reference Reagent Program (7, 8). Virus stocks were generated by transient transfection of 293T cells either with an IMCs or envelope deleted plasmid along with an envelope expression plasmid. BlaM-Vpr containing virus stocks were generated by also co-transfecting pMM310. Vpx-containing virus like particles were produced by the co-transfection of 293T cells with pSIV3^{+} and VSV-G expression plasmid. All virus containing supernatants were clarified using low speed centrifugation and filtered through a 0.45 um pore filters. BlaM-Vpr containing viruses and luciferase reporter viruses were pelleted through sucrose cushion gradients. The number of infectious particles was estimated on TZM-bl cells and capsid content of HIV-1 was determined by a p24^{gag} ELISA as previously described (9-11).

Infections. CD1a+ VEDCs, skin derived LCs and activated or non-activated lamina propria cells were exposed to virus at a multiplicity of infection (MOI) ranging from 0.1 – 15 as indicated. In the infections with protease inhibitor (Saquinavir 1μM), the protease inhibitor was maintained in

cultures at all times. Exposed cells were washed a minimum of 4 times to remove unbound virus. CD1a+ VEDCs and skin derived LCs were washed after 72 hours and lamina propria cells were washed after 2 hours of exposure. CD1a+ VEDCs, skin derived LCs and non-activated lamina propria cells were cultured in fresh RPMI complete, while activated lamina propria cells were cultured in RPMI complete with 20 u/ml of recombinant IL-2 (r-IL2). For the co-cultures, exposed and subsequently washed CD1a+ VEDCs were co-cultured with activated lamina propria cells in RPMI complete with 20 u/ml of r-IL2. Infections were monitored by removing around 50 ul of culture supernatant every 2 days and replacing with fresh media. Infectious virus concentration was estimated by infecting 1 X 10^4 TZM-bl cells with 8 serial two-fold dilutions of supernatant culture starting at 50 ul. All infections were done in duplicate in a 96 well format. Two days post-infection, TZM-bls were examined for beta-galactosidase production using Galacto-Light Plus System (Applied Biosystems). In every experiment, background level was estimated as the average of the relative light unit (RLU) from wells with TZM-bl alone plus 1 standard deviation. This background was subtracted from all wells, and wells with negative RLU were assigned a value of 0. Infections with protease inhibitor were monitored by estimating HIV RNA in culture supernatants. Briefly, RNA was isolated from culture supernatants using viral RNA isolation kit (Qiagen). HIV RNA was quantified using a previously published sensitive quantitative PCR assay with minor modifications (12).

Fusion Assay. Fusion assay was done as previously described (13). Around 5 X 10^4 to 1 X 10^5 CD1a+ VEDC were infected with BlaM-Vpr-containing virions (200 to 500 ng of $p24^{Gag}$) for 3 h at 37°C in 100 µl of RPMI medium. After infection, cells were then washed once with CO_2-independent RPMI medium and loaded with CCF2 dye (0.5 mM; Invitrogen). Cells were incubated for 18 h at room temperature in 200 µl of CO_2-independent RPMI medium supplemented with 10% FBS and 2.5 mM probenecid. Infected CD1a+ VEDCs were washed once in staining buffer (phosphate-buffered saline [PBS] with 2% FBS) and incubated for 30 min at room temperature with a CD1a directed antibody. The cells were next washed twice with PBS

containing 2% FBS. Cells were fixed with 100 µl 4% PFA for 30 min at RT. The change in emission fluorescence of CCF2 after cleavage by the BlaM-Vpr chimera was measured by flow cytometry with a LSRII flow cytometer (Becton Dickinson). Data were collected using FACSDiva software (Becton Dickinson) and analyzed with FlowJo software (TreeStar). The compensation was calculated after data collection on the basis of single-stain controls using FlowJo software.

Measuring HIV DNA intermediates. Late transcription products and integrated proviruses were measured as detailed previously (14). Briefly, DNA from virus exposed cells was isolated using Qiagen DNA Isolation kit. Late reverse transcription and integration products were quantified using quantitative PCR with primers and probes that anneal to LTR – *gag* and human *Alu* – *gag* regions respectively as described previously (14, 15). The number of HIV copies was determined using pNL4-3 as a standard. HIV molecules were normalized to copies per cell by albumin quantitative PCR. All samples were measured 3 independent times.

Transcription assay. CD1a+ VEDCs were exposed to 200ng of $p24^{gag}$ virus inoculums in the presence or absence of 10uM Maraviroc (MVC), a CCR5 inhibitor or 10uM AMD3100, a CXCR4 blocker. Pseudovirions incorporated the various envelopes with envelope deleted Lai and *luciferase* gene in the place of viral accessory gene *nef*. After 3 days, luciferase activity was measured, in duplicate, using Bright-Glo Luciferase Assay System (Promega) in the cultures.

HIV DNA quantitation and envelope sequences. Cells were isolated from HIV-1 infected vaginal tissue and lamina propria using similar techniques as above. Peripheral blood mononuclear cells (PBMCs) were isolated using Ficoll Hypaque density centrifugation. Quantitative PCR was used to estimate number of HIV-1 DNA copies and number of cells as previously described (12). All estimations were done in triplicate a minimum of 2 independent times. Envelope sequences were isolated using single genome amplification as described previously (16). Sequences were aligned using HIVAlign (https://www.hiv.lanl.gov/content/sequence/VIRALIGN/viralign.html) and alignments were manually edited in BioEdit. Maximum likelihood tree was generated using PhyML

(https://www.hiv.lanl.gov/content/sequence/PHYML/interface.html) with HKY85 model. Color and symbol coded phylogenetic tree was generated using RainbowTree (https://www.hiv.lanl.gov/content/sequence/RAINBOWTREE/rainbowtree.html). All unique sequences reported in this publication have been submitted to Genbank (accession numbers MG940909-MG940950). Envelopes were incorporated into an envelope deficient NL4-3 HIV-1 backbone using yeast gap-repair homologous recombination as described previously (16). Virus stocks were generated from 293T transfection and subsequent PBMC passage. Co-receptor usage was determined as described previously (16). Briefly, TZM-bls were exposed to virus stocks in the presence or absence of co-receptor inhibitors. Statistically significant difference in infection levels in the presence as compared to absence of inhibitors was used to determine the co-receptor usage. All conditions were tested in triplicate, and all assays were conducted with internal controls YU2 (R5), NL4-3 (X4), and 89.6 (R5X4).

T cell receptor re-arrangement quantitative assay. Cell preparations were assessed for frequency of re-arranged TCRG using a previously described quantitative PCR assay (17). Briefly, quantitative PCR was conducted with a proprietary mix of 10 ul of unlabeled TCRG primers (Invivoscribe), 10 ul of SYBR Green Power Up Master Mix (Invitrogen), and 5 ul of DNA from various cell preparations in a 25 ul reaction (18). Quantitative PCR standards consisted of serially diluted DNA from known quantity of magnetic bead isolated CD4+ T cells. Reaction conditions were as follows: 95°C holding stage for 7 minutes, followed by 45 cycles at 95°C for 45 seconds, 60°C for 1 minute, and 72°C for 90 seconds. All samples were tested in triplicate along with skin derived Langerhans cell preparations (negative control) and CD4+ T cells (positive control). Number of re-arranged TCRGs were normalized to cell copies as assessed by albumin quantitative PCR.

Supplemental Table 1. Characteristics of HIV-1 infected patients that provided vaginal tissue

Subject	Age (years)	Years with plasma virus level < 400	HIV - 1 medications	HIV copies per 1E6 CD1a+ VEDCs	HIV copies per 1E6 Lamina propria cells	HIV copies per 1E6 PBMCs
I	60	~ 6	TRV/EFV	311	NA	1261
II	62	~ 16	TRV/ZDV/RAL	191	2291	1561

TRV: Tenofovir disoproxil fumarate and emtricitabine
EFV: Efavirenz
ZDV: Zidovudine
RAL: Raltegravir
NA: not available

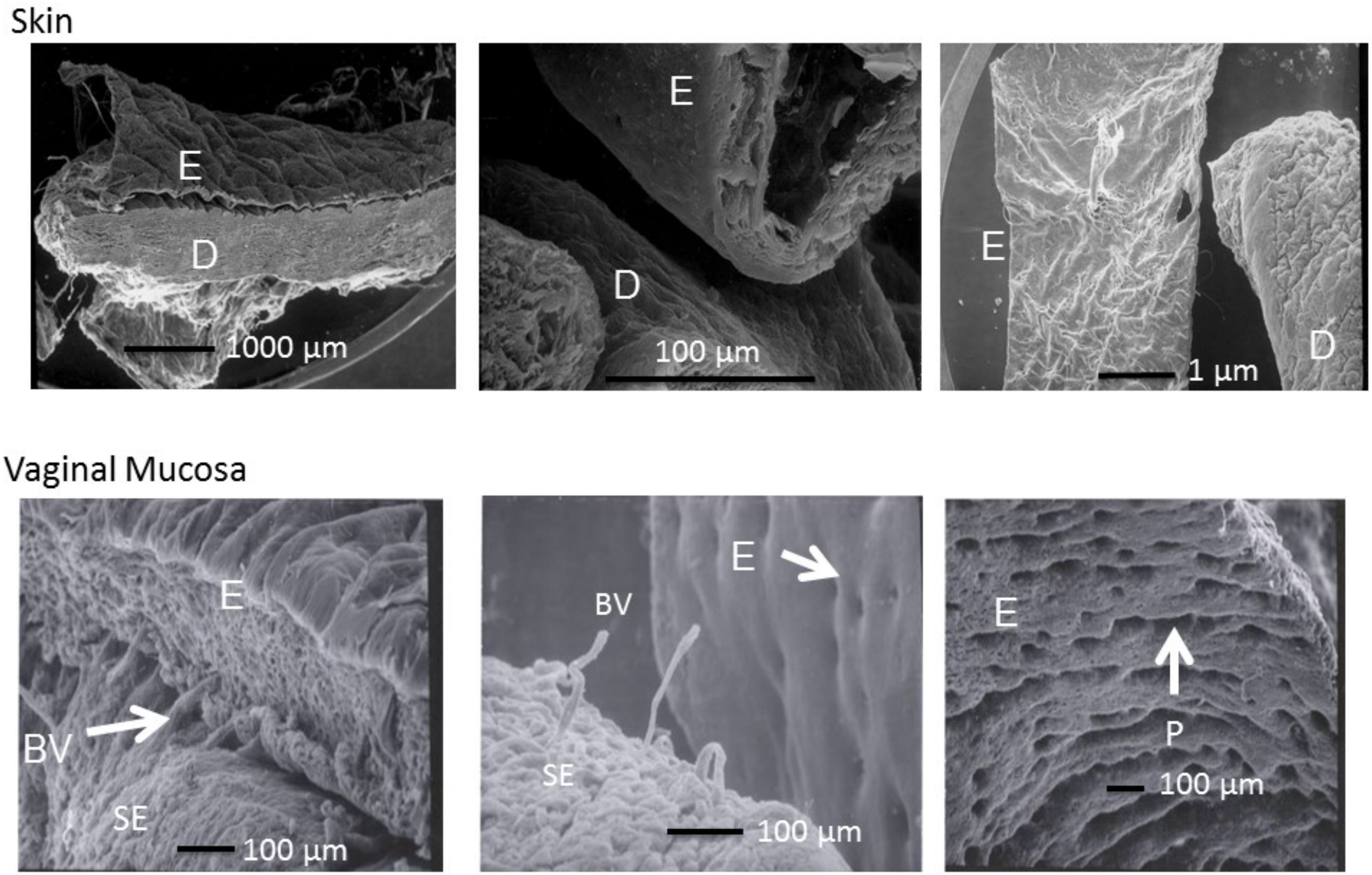

Supplemental Figure 1. Scanning transmission electron microscope images of skin (top) and vaginal mucosa (bottom). Mechanical separation of epithelium (E) from the sub-epithelium (SE) and dermis (D). Vaginal mucosa images show blood vessels (BV) and papilla (P) within the epithelium (E). Size of the black bars and adjacent numbers denote the magnification.

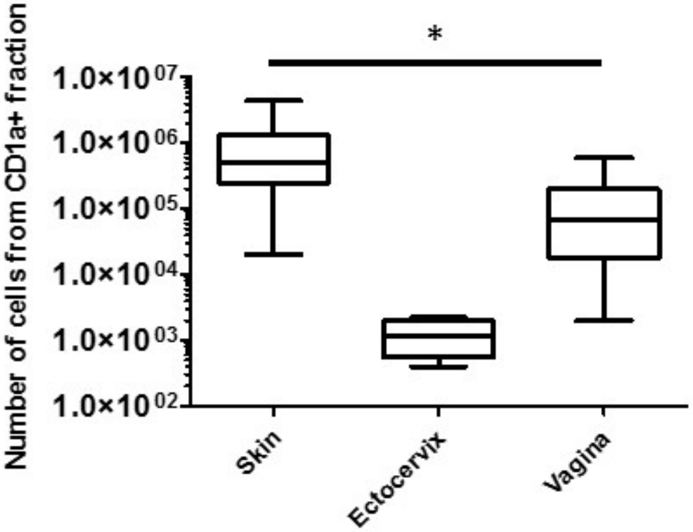

Supplemental Figure 2. Cell quantities obtained from different human tissues. Box plot showing cell quantities obtained from different discarded tissues. Cells quantities obtained from skin of discarded breast tissue (median $5.0X10^{5}$, range $2.0X10^{4}$ - $4.4X10^{6}$ cells) (n = 38), ecto-cervical tissue (median 1150, range 400 - 2200 cells) (n = 3), and vaginal tissue (median $6.8X10^{4}$, range $2.0X10^{3}$ - $6.0X10^{5}$ cells) (n = 47). Median shown by line in middle of box plot. Asterisk signifies significant difference ($p < 0.0001$, Wilcoxon rank sum test).

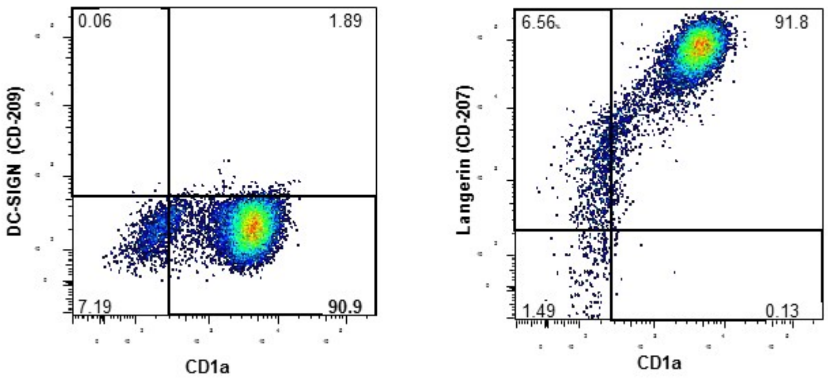

Supplemental Figure 3. Flow cytometric analysis of skin derived Langerhans cells. Representative example of cells isolated from skin tissue using discontinuous Opti-Prep gradients and CD1a conjugated magnetic beads. The skin was obtained from women undergoing reduction mammoplasties. Cells were stained for CD1a-FITC, DC-SIGN (CD-209), and langerin (CD-207). Numbers in the quadrants show % cells positive. Experiment has been conducted more than 10 times (2).

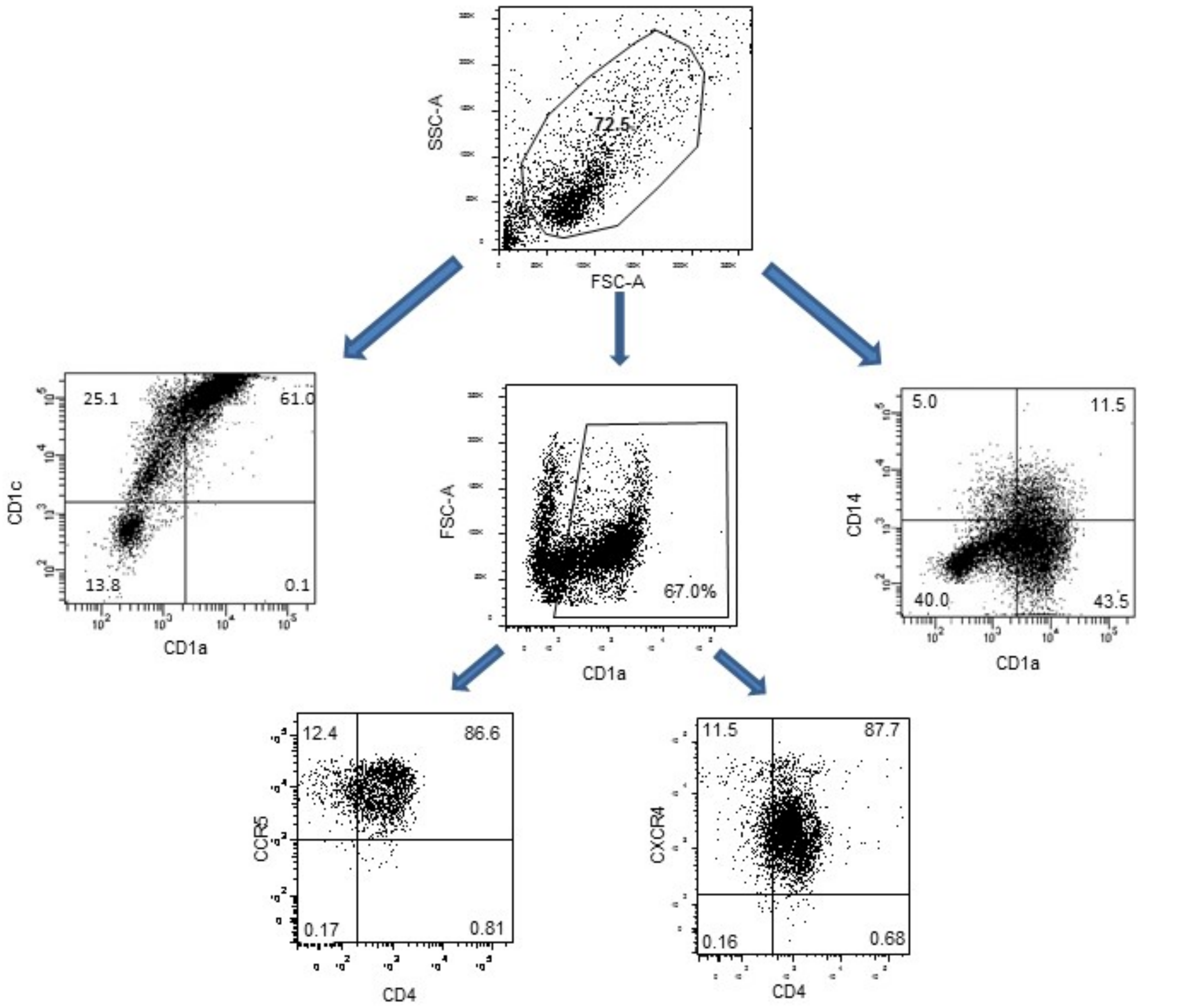

Supplemental Figure 4. Majority of CD1a+ VEDCs lack macrophage marker CD14. Representative dot-plots show staining for CD1a along with CD1c (left) and CD14 (right). Dot blots on the bottom show co-expression of CD4 either with CCR5 (left) or CXCR4 (right) in the CD1a+ cells. Numbers in the quadrants show % cells positive. This analysis was done in 3 different cell preparations.

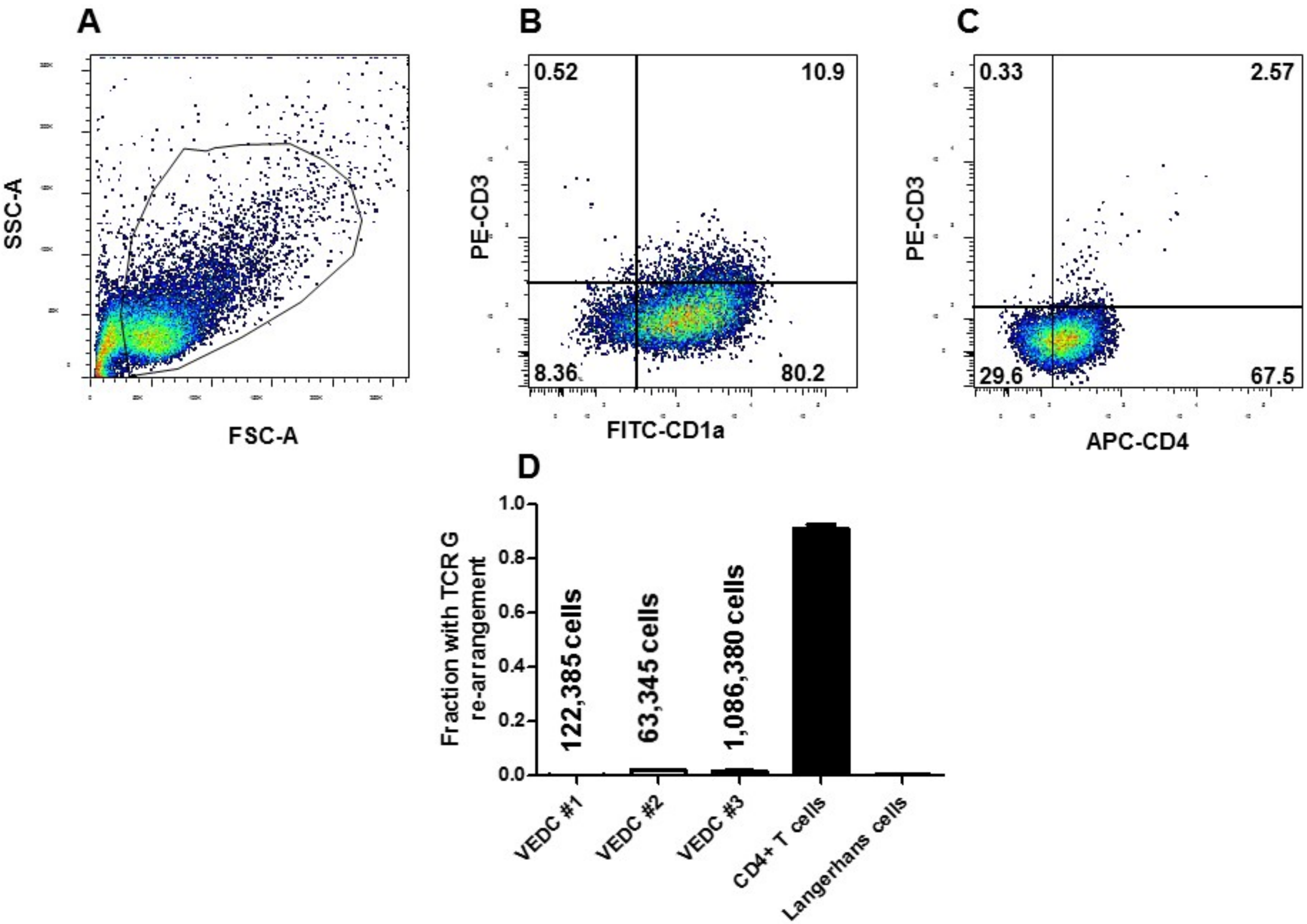

Supplemental Figure 5. Flow cytometric characterization for the presence of CD3+ CD4+ cells. Representative flow cytometric analysis among the isolated cells (A) that were CD3+ CD1a+ cells (B) and CD3+ CD4+ cells (C). Numbers in the quadrants show % cells positive. Experiments were done in 2 different cell preparations. Graph D shows the fraction of cells with T cell receptor G (TCR G) re-arrangement in 3 different CD1a VEDC preparations (VEDC #1 - #3), CD4+ T cells, and skin derived Langerhans cells. The total number of cells assessed in each of the 3 VEDC preparations is also shown above each bar.

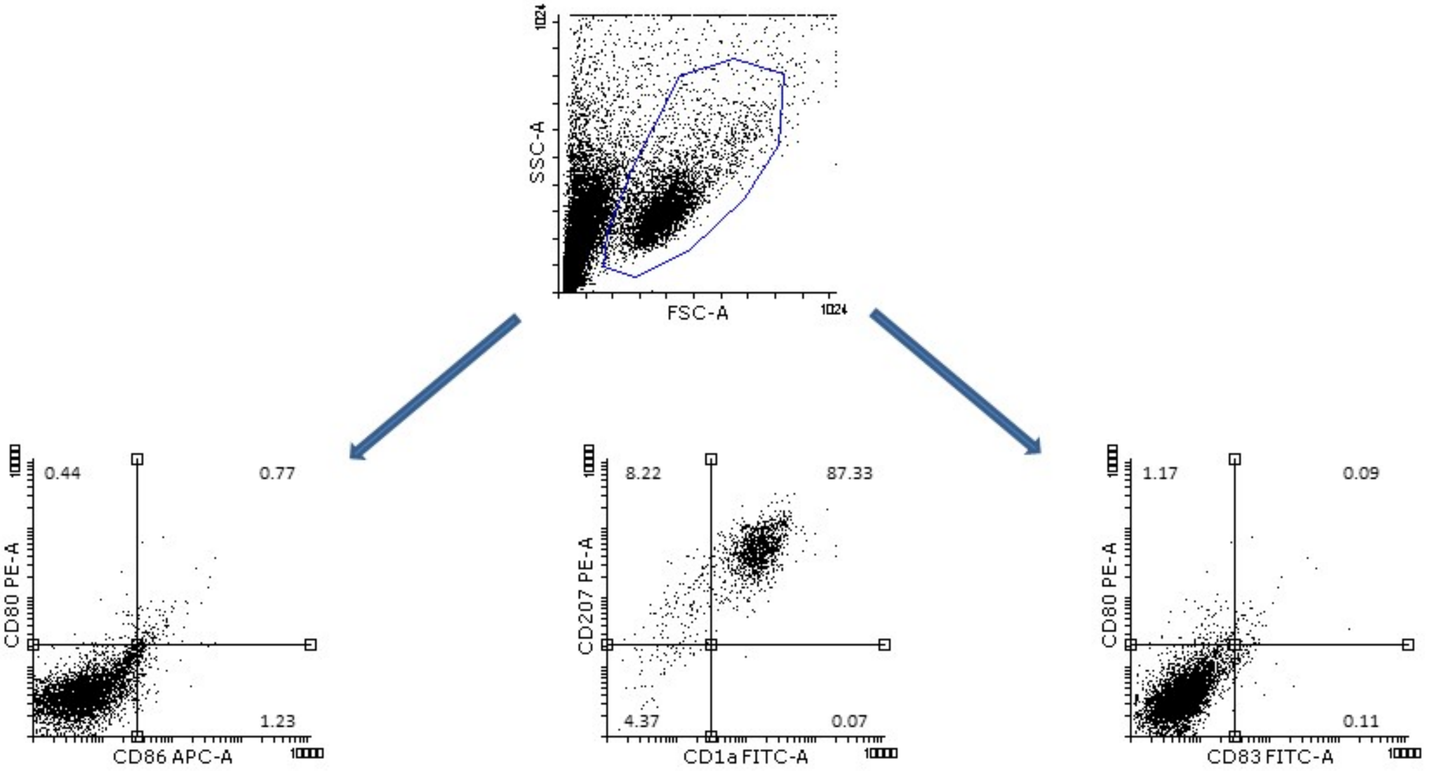

Supplemental Figure 6. Flow cytometric characterization of CD1a+ VEDCs activation state. Representative flow cytometric analysis of activation marker expression (CD80, CD86, and CD83) in CD1a+, CD207+ VEDCs. Numbers in the quadrants show % cells positive. Analysis was done using flowing software (version 2.5.1). Experiments were done in 3 different cell preparations.

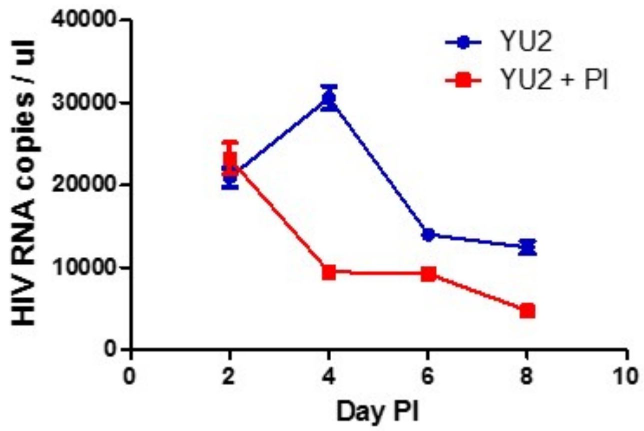

Supplemental Figure 7. Spreading infection observed in CD1a+ VEDC cultures in the absence but not the presence of protease inhibitor. CD1a+ VEDCs were exposed to CCR5-using virus (YU2) at a MOI of 10 in the presence (red) or absence (blue) of 1 µM Saquinavir, a protease inhibitor (PI). Protease inhibitors inhibit virus production by blocking a post-integration stage in the life cycle. Replication was monitored by estimating HIV RNA copies in the virus supernatant (y-axis). Days PI (x-axis) was defined as days after virus exposed cells were washed to remove unbound virus. Values are from 3 replicates and bars show standard error mean (SEM).

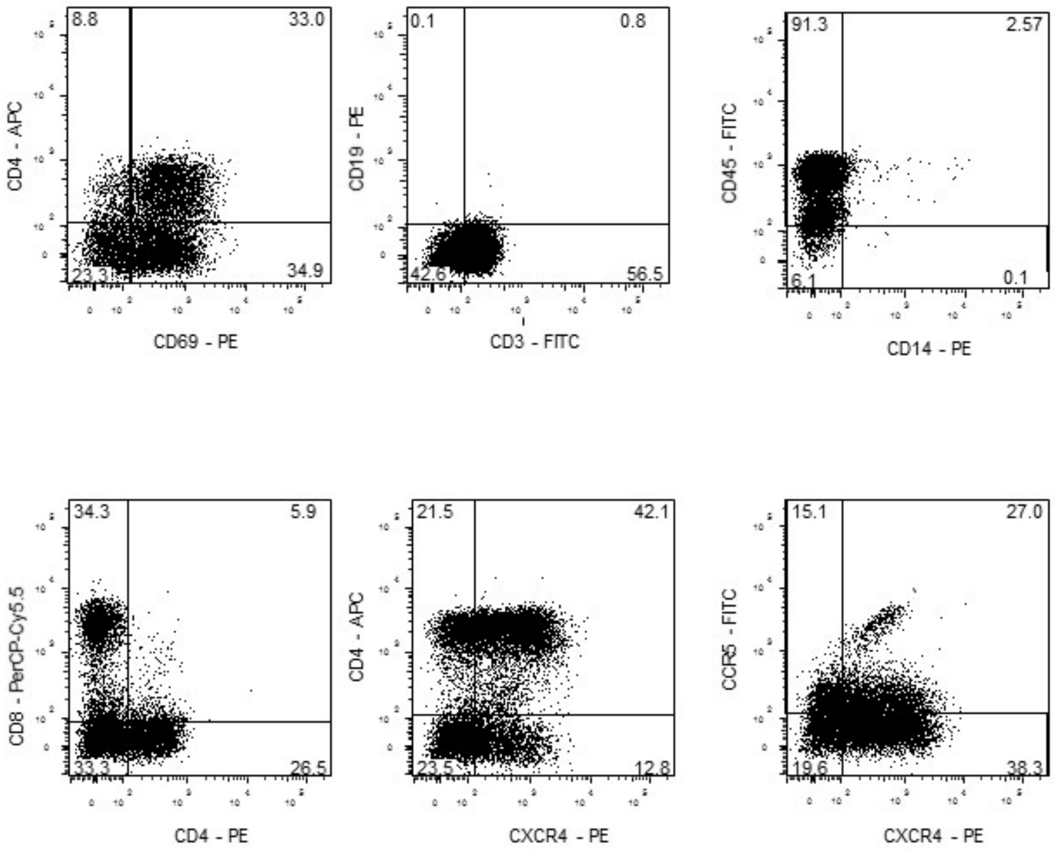

Supplemental Figure 8. Flow cytometric characterization of activated cells derived from the vaginal tissue lamina propria. Activated cells from the lamina propria are predominantly CD69+, CD3+, CD45+, and CD4 or CD8 positive lymphocytes that also express CCR5 and CXCR4. CD69 is a distinguishing marker for tissue resident lymphocytes. They are CD19 and CD14 negative suggesting they are not B cells or macrophages. Numbers indicate percent cells in each quadrant. Due to cell number limitations, all cells are not from the same donor. Staining was done in cells from 4 different tissues.

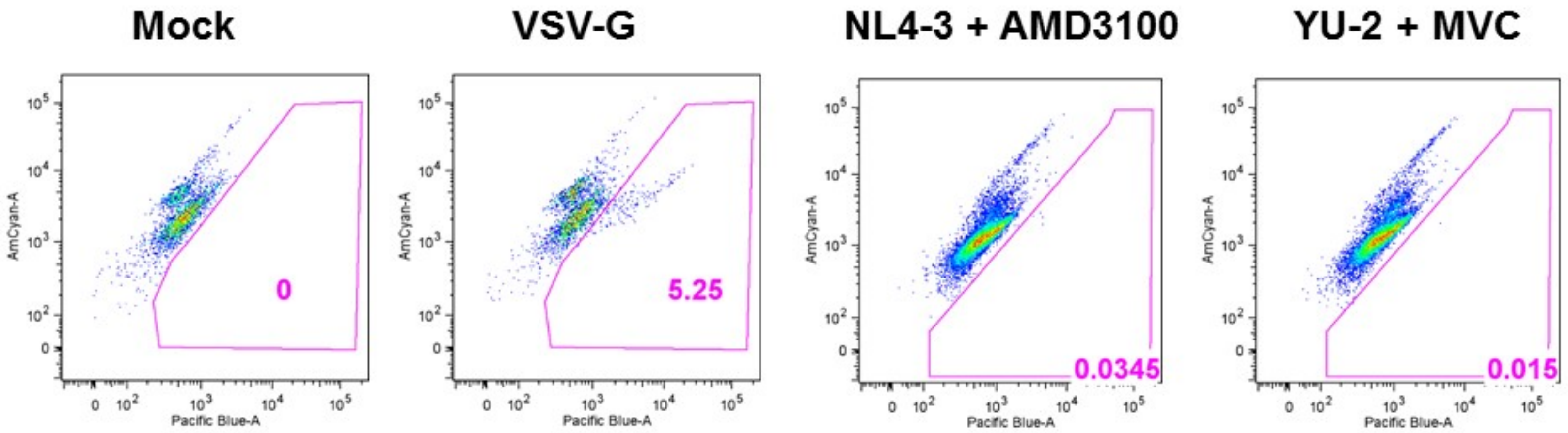

Supplemental Figure 9. HIV fusion, reverse transcription and integration in CD1a+ VEDCs. Figures show observed fusion in mock infected cells (negative control), and cells exposed to viruses pseudotyped with VSV-G (positive control) envelope, NL4-3 envelope with CXCR4 inhibitor, AMD3100, YU-2 envelope with CCR5 inhibitor, MVC. In each case, FACS analysis was restricted to the CD1a+ cells. Numbers in quadrant show percent of cells with observed fusion.

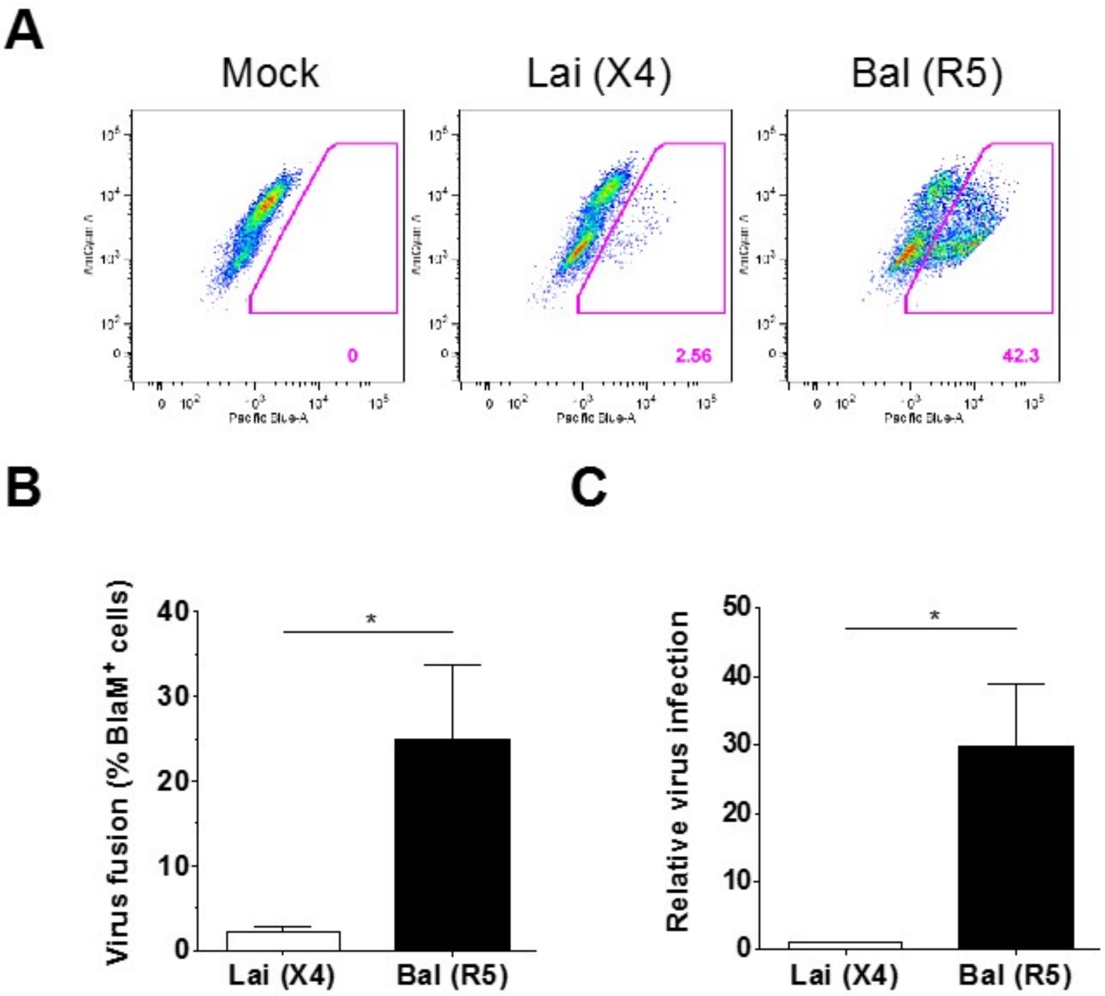

Supplemental Figure 10. Restricted infection of X4-tropic HIV-1 in immature monocyte-derived DCs (MDDCs) is primarily due to a block at virus entry. (A) Representative FACS profiles of immature MDDCs infected with BlaM-Vpr containing HIV-1 pseudotyped with Lai (X4) or Bal (R5) Env. **(B)** The percentage of $BlaM^{+}$ immature MDDCs from multiple infections with HIV-1 pseudotyped with Lai (X4) or Bal (R5) Env and the means ± SEM are shown (Lai, n=7, Bal, n=6). **(C)** Infection of immature MDDCs with HIV-1 pseudotyped with Lai (X4) or Bal (R5) Env. HIV-1 infection was quantified 2 days post infection by measuring luciferase activity in cell lysates and values were normalized to those of Lai (X4), and the means ± SEM are shown (n=5). Two-tailed p values were calculated using **(B)** unpaired t-test or **(C)** one sample t-test in GraphPad Prism 5. *: $p<0.05$.

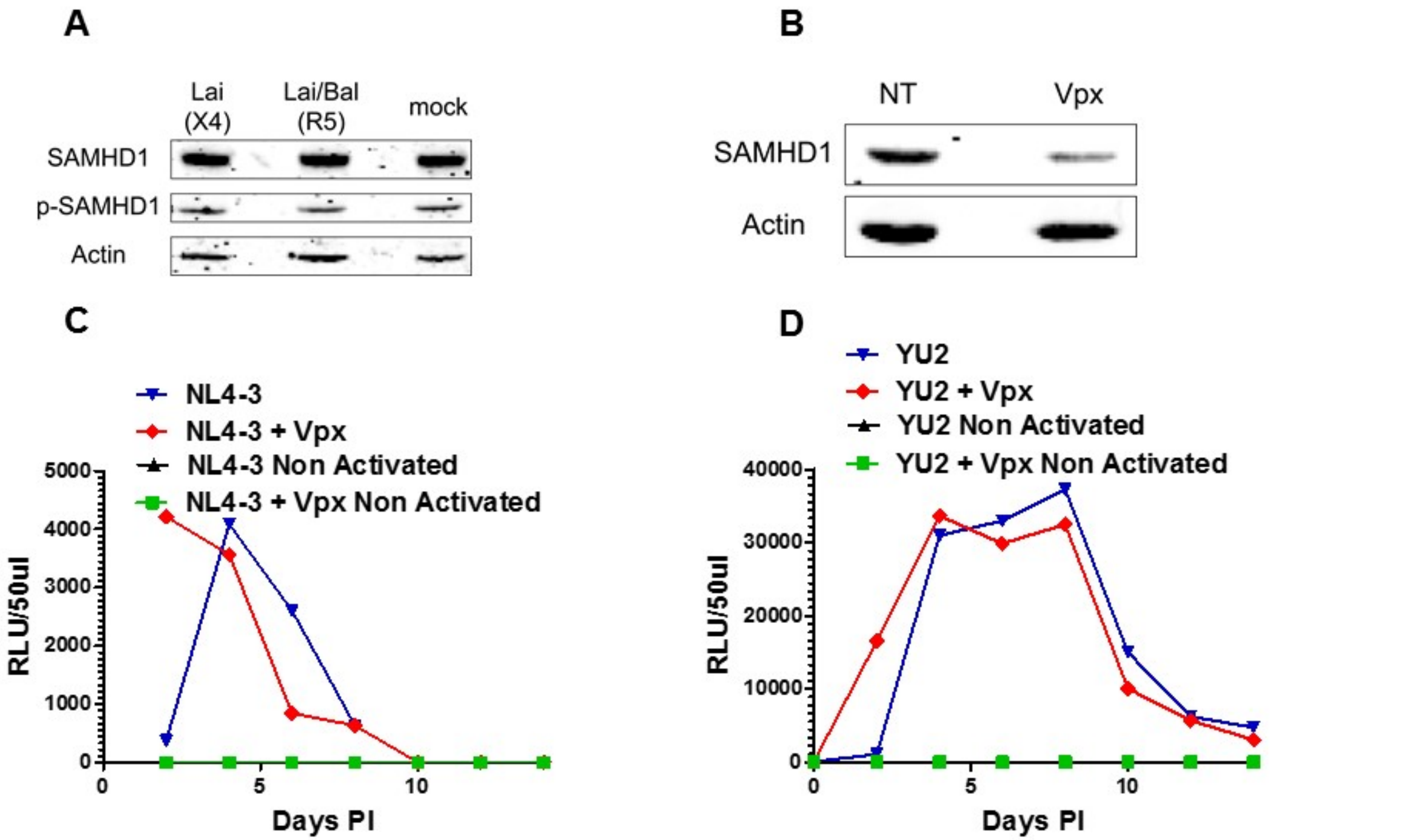

Supplemental Figure 11. SAMHD1 levels and impact on virus infection. Total and inactive phosphorylated form of SAMHD1 expression levels do not change after virus exposure **(A)** but levels decrease after Vpx exposure **(B)**. **(A)** Whole cell lysates from CD1a+ VEDCs exposed to Lai with Lai-env (X4), Lai with Bal-env (R5), and no virus (mock) were separated by SDS-PAGE and analyzed for total SAMHD1, phosphorylated SAMHD1 (p-SAMHD1), and actin (loading control). **(B)** Whole cell lysates from CD1a+ VEDCs incubated with SIVmac Vpx containing virus like particles (VLPs) (Vpx) or untreated (NT) were separated by SDS-PAGE and analyzed for total SAMHD1 and actin (loading control). **(C and D)** Observed replication of NL4-3 (MOI 0.1) **(C)** and YU2 (MOI 1.0) **(D)** in activated (blue and red) and non-activated (black and green) lamina propria lymphocytes in the presence (red and green) or absence (blue and black) of Vpx containing VLPs. Replication in **(C and D)** was examined in cells from different donors.

REFERENCES

1. Pena-Cruz, V., Ito, S., Oukka, M., Yoneda, K., Dascher, C.C., Von Lichtenberg, F., and Sugita, M. 2001. Extraction of human Langerhans cells: a method for isolation of epidermis-resident dendritic cells. *J Immunol Methods* 255:83-91.
2. Pena-Cruz, V., Etemad, B., Chatziandreou, N., Nyein, P.H., Stock, S., Reynolds, S.J., Laeyendecker, O., Serwadda, D., Gray, R.H., Lee, S., et al. 2013. HIV-1 envelope replication and α4β7 utilization among newly infected subjects and their corresponding heterosexual partners. *Retrovirology* 10:162.
3. Kashihara-Sawami, M., Horiguchi, Y., Ikai, K., Takigawa, M., Ueda, M., Hanaoka, M., and Imamura, S. 1988. Letterer-Siwe disease: immunopathologic study with a new monoclonal antibody. *J Am Acad Dermatol* 18:646-654.
4. Akiyama, H., Ramirez, N.G., Gudheti, M.V., and Gummuluru, S. 2015. CD169-mediated trafficking of HIV to plasma membrane invaginations in dendritic cells attenuates efficacy of anti-gp120 broadly neutralizing antibodies. *PLoS Pathog* 11:e1004751.
5. Hatch, S.C., Archer, J., and Gummuluru, S. 2009. Glycosphingolipid composition of human immunodeficiency virus type 1 (HIV-1) particles is a crucial determinant for dendritic cell-mediated HIV-1 trans-infection. *J Virol* 83:3496-3506.
6. Goujon, C., Jarrosson-Wuilleme, L., Bernaud, J., Rigal, D., Darlix, J.L., and Cimarelli, A. 2006. With a little help from a friend: increasing HIV transduction of monocyte-derived dendritic cells with virion-like particles of SIV(MAC). *Gene Ther* 13:991-994.
7. Owen, S.M., Ellenberger, D., Rayfield, M., Wiktor, S., Michel, P., Grieco, M.H., Gao, F., Hahn, B.H., and Lal, R.B. 1998. Genetically divergent strains of human immunodeficiency virus type 2 use multiple coreceptors for viral entry. *J Virol* 72:5425-5432.

8. Levy, J.A., Hoffman, A.D., Kramer, S.M., Landis, J.A., Shimabukuro, J.M., and Oshiro, L.S. 1984. Isolation of lymphocytopathic retroviruses from San Francisco patients with AIDS. *Science* 225:840-842.

9. Sagar, M., Wu, X., Lee, S., and Overbaugh, J. 2006. HIV-1 V1-V2 envelope loop sequences expand and add glycosylation sites over the course of infection and these modifications affect antibody neutralization sensitivity. *J Virol* 80:9586-9598.

10. Kimpton, J., and Emerman, M. 1992. Detection of replication-competent and pseudotyped human immunodeficiency virus with a sensitive cell line on the basis of activation of an integrated beta-galactosidase gene. *J Virol* 66:2232-2239.

11. Sagar, M., Akiyama, H., Etemad, B., Ramirez, N., Freitas, I., and Gummuluru, S. 2012. Transmembrane Domain Membrane Proximal External Region but Not Surface Unit-Directed Broadly Neutralizing HIV-1 Antibodies Can Restrict Dendritic Cell-Mediated HIV-1 Trans-infection. *J Infect Dis* 205:1248-1257.

12. Malnati, M.S., Scarlatti, G., Gatto, F., Salvatori, F., Cassina, G., Rutigliano, T., Volpi, R., and Lusso, P. 2008. A universal real-time PCR assay for the quantification of group-M HIV-1 proviral load. *Nat Protoc* 3:1240-1248.

13. Cavrois, M., De Noronha, C., and Greene, W.C. 2002. A sensitive and specific enzyme-based assay detecting HIV-1 virion fusion in primary T lymphocytes. *Nat Biotechnol* 20:1151-1154.

14. Zhong, P., Agosto, L.M., Ilinskaya, A., Dorjbal, B., Truong, R., Derse, D., Uchil, P.D., Heidecker, G., and Mothes, W. 2013. Cell-to-cell transmission can overcome multiple donor and target cell barriers imposed on cell-free HIV. *PLoS One* 8:e53138.

15. Agosto, L.M., Hirnet, J.B., Michaels, D.H., Shaik-Dasthagirisaheb, Y.B., Gibson, F.C., Viglianti, G., and Henderson, A.J. 2016. Porphyromonas gingivalis-mediated signaling through TLR4 mediates persistent HIV infection of primary macrophages. *Virology* 499:72-81.

16. Etemad, B., Ghulam-Smith, M., Gonzalez, O., White, L.F., and Sagar, M. 2015. Single genome amplification and standard bulk PCR yield HIV-1 envelope products with similar genotypic and phenotypic characteristics. *J Virol Methods* 214:46-53.

17. DiNapoli, S.R., Ortiz, A.M., Wu, F., Matsuda, K., Twigg, H.L., III, Hirsch, V.M., Knox, K., and Brenchley, J.M. 2017. Tissue-resident macrophages can contain replication-competent virus in antiretroviral-naive, SIV-infected Asian macaques. *JCI Insight* 2.

18. Yao, R., and Schneider, E. 2007. Detection of B- and T-cell-specific gene rearrangements in 13 cell lines and 50 clinical specimens using the BIOMED-2 and the original InVivoScribe primers. *Leuk Lymphoma* 48:837-840.
